# A qualitative study to identify critical attributes and attribute-levels for a discrete choice experiment on oral pre-exposure prophylaxis (PrEP) delivery among young people in Cape Town and Johannesburg, South Africa

**DOI:** 10.1186/s12913-020-05942-8

**Published:** 2021-01-06

**Authors:** Janan J. Dietrich, Millicent Atujuna, Gugulethu Tshabalala, Stefanie Hornschuh, Mamakiri Mulaudzi, Michelle Koh, Nadia Ahmed, Richard Muhumuza, Andrew S. Ssemata, Kennedy Otwombe, Linda-Gail Bekker, Janet Seeley, Neil A. Martinson, Fern Terris-Prestholt, Julie Fox

**Affiliations:** 1 Perinatal HIV Research Unit (PHRU), Faculty of Health Sciences, University of the Witwatersrand, Johannesburg, South Africa; 2 Health Systems Research Unit, South African Medical Research Council, Bellville, South Africa; 3 Desmond Tutu HIV Centre, University of Cape Town, Cape Town, South Africa; 4 Harvard Global Health Institute, Harvard University, Cambridge, MA USA; 5 Mortimer Market Centre, Central North West London NHS Trust, Off Caper Street, London, WC1E 6 JB UK; 6 Medical Research Council/Uganda Virus Research Institute , Entebbe, Uganda; 7 Department of Global Health and Development, London School of Hygiene and Tropical Medicine, London, UK; 8 King’s College London, London, UK

**Keywords:** Discrete choice experiment (DCE), Conjoint analysis, HIV, Oral pre-exposure prophylaxis (PrEP), Preferences, Adolescents, Young people, South Africa

## Abstract

**Background:**

The uptake and adherence of daily oral PrEP has been poor in high-risk populations in South Africa including young people. We used qualitative research methods to explore user preferences for daily and on-demand oral PrEP use among young South Africans, and to inform the identification of critical attributes and attribute-levels for quantitative analysis of user preferences, i.e. a discrete choice experiment (DCE).

**Methods:**

Data were collected between September and November 2018 from eight group discussions and 20 in-depth interviews with young people 13 to 24 years in Cape Town and Johannesburg. Using a convenience sampling strategy, participants were stratified by sex and age. Interviewers used a semi-structured interview guide to discuss several attributes (dosing regimen, location, costs, side effects, and protection period) for PrEP access and use. Group discussions and in-depth interviews were audio-recorded, transcribed verbatim and translated to English. We used framework analysis to explore context-specific attributes and attribute-levels for delivering oral PrEP in South Africa. The adolescent community advisory board, expert and study team opinions were consulted for the final DCE attributes and levels.

**Results:**

We enrolled 74 participants who were 51% (*n* = 38/74) male, had a median age of 18.5 [Interquartile range = 16–21.25] years, 91% (*n* = 67/74) identified as heterosexual and 49% (*n* = 36/74) had not completed 12th grade education. Using the qualitative data, we identified five candidate attributes including (1) dosing regimen, (2) location to get PrEP, (3) cost, (4) route of administration and (5) frequency. After discussions with experts and the study team, we revised the DCE to include the following five attributes and levels: dosing regime: daily, and on-demand PrEP; location: private pharmacy, public clinic, mobile clinic, ATM); cost: free-of-charge, R50 (~2GBP), R265 (~12GBP); side effects: nausea, headache, rash; and duration of protection: fulltime protection versus when PrEP is used).

**Conclusions:**

There is limited literature on qualitative research methods describing the step-by-step process of developing a DCE for PrEP in adolescents, especially in resource-constrained countries. We provide the process followed for the DCE technique to understand user preferences for daily and on-demand oral PrEP among young people in South Africa.

**Supplementary Information:**

The online version contains supplementary material available at 10.1186/s12913-020-05942-8.

## Background

The prevention of Human Immunodeficiency Virus (HIV) in young people in South Africa remains a priority, firstly because it is a preventable chronic condition which would in turn negate the risk of associated physical and psychological co-morbidities, and secondly because of sustained high HIV prevalence and incidence among adolescents in both South Africa and sub-Saharan Africa (SSA). Moreover, interventions for HIV prevention within SSA have had limited effect, despite high levels of HIV awareness. The limited effect of HIV prevention interventions is largely attributable to poor adherence observed in major clinical trials [1–7].

Globally, pre-exposure prophylaxis (PrEP) shows efficacy in preventing HIV amongst males and females [8–10], with a daily PrEP regimen shown to be effective and associated with decreasing HIV incidence in heterosexual males and females, males who have sex with males (MSM), transgender females, and people who inject drugs (PWID) [2, 9, 11–13]. This regimen has subsequently been recommended by the World Health Organization (WHO) for individuals at high risk for infection [2]. On-demand PrEP, which consists of four pericoital doses, represents a potentially more cost-effective approach [14]. However, the on-demand regimen is not yet recommended for populations other than MSM due to the lack of studies and the complexity of the regimen [15].

Despite availability of PrEP in the public sector to females 15–24 years in South Africa [16] there is still a lack of awareness and uptake of PrEP among young South Africans [17]. The expansion of PrEP is urgently required across SSA, but can only be optimised by understanding barriers to PrEP uptake in young people. Currently, there is no model of PrEP provision for adolescent males who have been over-looked in HIV prevention research [15] but contribute to HIV incident infections [18].

Discrete choice experiments (DCEs), also known as conjoint analysis, can direct researchers to user needs, and inform how to best formulate or implement a health intervention, as DCE allows researchers both to identify attributes [19] of an intervention that make it attractive and also to rank the importance of that attribute [20]. The results from DCEs are critical in predicting the success of different approaches to implementing particular healthcare interventions in relevant communities or populations [21, 22].

DCE-based studies on PrEP preferences in specific cohorts are lacking, including young people in Africa. Recently, Dubov et al. (2019) performed a DCE to inform efforts to improve PrEP access and uptake among MSM in the United States [23]. As for DCE-based studies on PrEP in the African context, Kuteesa et al. (2019) conducted a DCE-based study investigating fishing communities in Uganda [24], while Lancaster et al. (2019) examined preferences among female sex workers (FSWs) in Malawi [25]. There has also been an increased interest in using DCEs to explore stakeholder preferences for healthcare interventions and changes in policy, as well as using them to support the prioritization, design and implementation of healthcare interventions [20].

The Combined HIV Adolescent Prevention Study (CHAPS) aims to investigate the acceptability and feasibility of implementing daily and on-demand PrEP among adolescents in South Africa, Uganda and Zimbabwe and to determine an on-demand PrEP dosing schedule for insertive sex in young males [26]. As part of the social science component, we used qualitative research to develop DCE choice sets to identify attributes and attribute levels for PrEP delivery among young people in South Africa.

The DCE technique can be simplified into four components: survey design, piloting and administration to participants, data analysis, and implement interventions for the relevant populations, or design further experiments [20, 27]. In this paper we focus on the preliminary qualitative research conducted to design the DCE for survey admistration.

Therefore, the aim of this paper is to describe qualitative research methods used to identify and develop the DCE choice sets and the salient attributes and preferences for PrEP delivery in young people aged 13–24 years in two South African cities.

## Methods

### Study design

A qualitative research approach was selected to form part of a broader rigorous DCE approach, wherein the qualitative research is good practice in the formative stage to develop and design the DCE [22]. Data collection from in-depth interviews (IDIs) allowed for individual perspectives while group discussions (GDs) provided data collection within the context of a larger group [28].

### Study setting

Data were collected from September to November 2018 at the Perinatal HIV Research Unit (PHRU) in Johannesburg and the Desmond Tutu HIV Centre (DTHC) in Cape Town. Participants were recruited in informal peri-urban communities established as residential areas for black South Africans during apartheid, characterized by overcrowding, unemployment, with limited resources and service delivery [29].

The PHRU is situated at the Chris Hani Baragwanath Academic Hospital in Soweto, has conducted research in HIV prevention, care and treatment for more than 20 years as well as providing healthcare services for the population of Soweto. Soweto is southwest of Johannesburg city in the province of Gauteng, and is estimated to have a population of ~ 2.5–3 million in an area of 63 km^2^ [30]. The prevalence of HIV in the Gauteng province is 15.2% among 15–49 year olds [31]. The second site, the DTHC is located in the Klipfontein/Mitchells Plain sub-district, in the Southern part of the Cape Town Metro. Compared to other district municipalities within the Western Cape, the Cape Town Metro bares the highest population size and prevalence of HIV [31]. Both PHRU and DTHC have a well established Adolescent Community Advisory Boards (ACAB), who are deeply rooted in their respective communities.

### Initial literature review

A literature search was conducted to start the attribute development process using the terms in Discrete choice experiment (DCE), Sub-saharan Africa (SSA), South Africa (SA), pre-exposure prophylaxis (PrEP), young people. The terms were used in a variety of combinations. While there has been a DCE-based study that included preferences concerning PrEP in South Africa [21], this study conducted in Johannesburg examined a variety of other HIV and pregnancy prevention methods along with PrEP, and focused only on female South Africans in diverse settings. For our study, based on literature, important attributes for delivering PrEP to young people included the cost of PrEP, the location of PrEP dispensaries, and dosage frequency.

### Procedures of the qualitative study for developing the DCE

As part of the larger CHAPS study, qualitative data were collected between September to November 2018 through eight group discussions (GDs) and 20 in-depth interviews (IDIs) with young people aged 13 to 24 years; four GDs and 10 IDIs in Cape Town and Johannesburg, respectively. In total, 54 participants took part in GDs; 28 in Johannesburg and 26 in Cape Town and a total of 20 participated in IDIs.

### Study population, sampling and recruitment process

Fieldworkers used a convenience sampling strategy to recruit eligible males and females 13–24 years, who were HIV negative on a rapid HIV test (Soweto used the ABON HIV 1/2/O Tri-Line Human Immunodeficiency Virus Rapid Test Device and Cape Town used the Alere Determine test) and reported sexual activity in the past 3 months. Most participants in our study had moderate to no prior knowledge about PrEP. In Soweto, participants for IDIs and GDs were actively recruited through community organisations, taxi ranks and outside premises of public meeting places such as shopping malls and community parks. In Cape Town, participants were recruited through local schools, shopping malls, the library, word of mouth referrals and walk in at the research clinic.

### Data collection procedures

IDIs and GDs were stratified by age (13–17 years and 18–24 years) and sex (males and females) for Soweto but not for Cape Town. GDs included mixed sex and age groups in Cape Town. Prior to the interviews, participants completed a short five-minute socio-demographic questionnaire [Additional File 1]. GDs and IDIs were facilitated by experienced and trained researchers fluent in English and local languages used (e.g. IsiZulu, IsiSesotho and IsiXhosa). They were conducted in secure and private locations at both sites. During the GDs, two researchers were present, one served as the main facilitator and the other as a note taker and co-facilitator. A semi-structured guide including open ended questions and relevant probes was used to facilitate and guide the discussions during the IDIs and GDs [Additional File 2]. Participants were allocated a participant identification number, which was used while completing the demographic questionnaire and during the GDs and IDIs. Participants used pseudonyms during all discussions to maintain anonymity. The IDIs and GDs lasted 36 min on and 100 min on average, respectively. All IDIs and GDs were audio-recorded, transcribed verbatim, and later translated to English.

### Measures

The brief socio-demographic questionnaire included questions on sex, age, race, primary home language, sexual orientation, and education level.

Trained qualitative researchers used a semi-structured interview guide to conduct the GDs and IDIs. The guide covered topics to identify several attributes for PrEP access and use, including: choice of dosing regimen (i.e. daily versus on-demand PrEP), alternative routes of PrEP administration (i.e. implant and injection), dispensing location (pharmacy, clinic, adolescent/youth centre, doctors office), and time to be spent in obtaining PrEP.

### Ethical approval

This study was approved by the Human Research Ethics Committees of the University of the Witwatersrand and University of Cape Town. Written study informed consent or assent was obtained from all participants. In Soweto, parents/ legal guardians provided written informed consent for participants younger than 18 years. Participants and parent/legal guardian signed separate forms for audio-recording. Cape Town had a parental waiver and did not require parental consent for participants under 13–17 years. Therefore, for Cape Town, participants provided written consent regardless of age. Participants were reimbursed R100 (~ 5GBP) in Johannesburg and R120 (~6GBP) in Cape Town respectively for their study participation.

### Data analysis

#### Qualitative data analysis

Framework analysis, a data analysis approach relevant for applied research, was used to analyse the data [32]. Framework analysis provides a highly systematic method of categorizing and organizing data according to key themes, concepts and emergent categories in grids or matrices. Transcripts were coded to identify context-specific attributes and attribute-levels for delivering oral PrEP in South Africa. Initial coding was conducted by two trained qualitative research team members at each site. Initially, the researchers read through four transcripts (two GDs and two IDIs) to gain an understanding of the data to develop an Excel spreadsheet codebook. Each transcript was then individually coded using a line by line technique to assign codes to text. A second analyst also coded the same transcripts using the line by line technique to identify codes. Both analysts then met to discuss codes identified and differences were discussed. If there was a disagreement among codes, this was discussed until a common agreement was reached Codes were then categorized into themes and sub-themes by both researchers. Themes identified were captured on an Excel spreadsheet and a comprehensive codebook was developed. The codebook was shared with the rest of the team from both sites for final review and input. In order to ensure data trustworthiness, analysis was conducted in a consistent manner: we used the same codebook across all sites, and conducted frequent comparison on code use through discussion across sites. The main analyst for this paper reviewed transcripts and coding for all sites to ensure that the coding was indeed aligned with transcripts.

#### Quantitative data analysis

Descriptive statistics and frequencies were used to analyse socio-demographic characteristics using Statistical Package for Social Sciences (SPSS) Version 25.0 [33].

## Results

Participant characteristics.

The median age of participants was 18.5 years (interquartile range [IQR = 16–21.25]), 51% (*n* = 38/74) were male, 91% (*n* = 67/74) of participants identified as heterosexual, all were Black Africans (*n* = 74/74, 100%), and 49% (*n* = 36/74) had not completed 12th grade education.

### Qualitative results to identify and develop attributes and attribute-levels

Table 1 illustrates the full list of all attributes and attribute-levels, which were identified in consensus among the two qualitative research analysts. Initially, eight candidate attributes with respective attribute levels were identified including (1) Frequency of administration, (2) side effects, (3) cost, (4) location of PrEP dissemination, (5) person responsible for dispensing, (6) duration of PrEP use, (7) pill intake, and (8) alternative routes of administration. Attributes and attribute-levels were identified from the transcripts and prominent participants’ quotes were directly extracted to illustrate each attribute and attribute-level (Table 1).

**Table 1:** List of DCE Attributes

Attribute label	Lay terminology	Key quotations from qualitative data	Labels of plausible levels	Frequency	Final inclusion
**Frequency of administration**	**How often one can take PrEP**	*“I prefer taking it [PrEP] daily because as I have said we do not plan to have sex so if I take it daily even if there is a certain day which chooses to come up for me to have sex I would know that I have taken the PrEP medication and I would know that I have protected myself even more now because I will not take an advantage of it”(IDI, Male,18–24 years, Soweto)*	**Daily**	**20**	**YES**
		*“I: And then you were saying that you would like to take PrEP when you are going to have sex? R: Yes. I: But then for how long now? R: For how long? I may maybe say I would take it [PrEP] 5 days before. If I know that on that weekend I am going to be seen by my partner. (IDI, Male, 13–17 years, Cape Town)*	**On demand**	**9**	
**Side Effects**	**Negative reaction to medication**	*“Sometimes you know when we vomit you get confused, you don’t know whether are you sick or you nauseous.” (IDI, Female,18–24 years, Soweto)*	**Nausea**	**7**	**YES**
		*“I occasionally had headaches” (IDI, Female, 18–24 years, Cape Town)*	**Headache**	**5**	
		*“I think that if there is a problem, it will help to show that there is a problem somewhere, you might get a rash and you might think that you have an allergy” (GD, Females,18–24 years, Soweto)*	**Rash**	**3**	
**Cost**	**How much to pay for PrEP**	*“I agree with those who said we get it [PrEP] for free, some families are struggling financially and I live with my grandmother which means that from my granny’s money I have to get money for PrEP?” (GD, Females,18–24 years, Soweto)*	**Free**	**6**	**YES**
		*“R: R50. I: Okay you would buy it [PrEP] for R50 [confirms]. I: And then how long would it [PrEP] last you? R: A month.” (IDI, Females, 18–24 years, Cape Town)*	**ZAR10–150** **(~ 50 penny – 7GBP)**	**22**	
		*“Maybe it [PrEP] could be, maybe 10 pills could be R250.” (IDI, Female, 13–17 years, Soweto)*	**ZAR200–300** **(~9–14 GBP)**	**3**	
**Location of dissemination**	**Where to get PrEP**	*“It will be easy for me to get it [PrEP] from the clinic because I don’t have money, I don’t have money to buy it, so that’s why I am saying the clinic. It will be easy for me to get it [PrEP] from the clinic.” (GD, Males,13–17 years, Soweto)*	**Clinic**	**25**	**YES**
		*“I would like to have it [PrEP] if available at the pharmacy and be sold there, like Shoprite [local low-cost store]. At Shoprite pharmacy no one will see you when you go there and no one knows you so you will just buy it. “(GD, Male and Female, 15–23 years, Cape Town)*	**Pharmacy**	**14**	
**Person responsible for dispensing PrEP**	**Who to give out PrEP**	*“Doctors who are treating HIV because they won’t just give you the pills, they will even explain why you must use something like that and how you must use it [PrEP]. (IDI, Male,13–17 years, Soweto)*	**Doctor**	**15**	**NO**
		*“A nurse, a female, but like around our age even if it’s 30, you see but someone who understands. (GD, Males and Females, 17–22 years, Cape Town)*	**Nurse**	**8**	
		*“I would prefer them [HIV counselors] because they are educated to counsel you first and make sure you are okay before you take something you don’t know. Nurses can explain but they don’t explain as well as a counsellor. A counsellor listens to you and you both listen to each other whereas a nurse just tells you what to do and they don’t even want to know about your concerns” (GD, Female, 18–24 years, Soweto)*	**HIV counselor**	**6**	
**Duration of PrEP use**	**Length of time to take PrEP**	*“So knowing that you taking PrEP for the rest of your life you’re safe you don’t have to worry about anything.” (IDI, Female, 18–24 years, Soweto)*	**Lifetime**	**11**	**NO**
		*“I would take it [PrEP] 5 days before … If I know that on that weekend I am going to be seen by my partner.”(IDI, Male,13–17 years, Cape Town)*	**Five days before sex**	**1**	
		*“It should be ten years just like the loop so that we can see how well people take to it [PrEP]. Like this thing that they put in your arm [refers to implant]” (GD, Females,18–24 years, Soweto)*	**Ten years**	**2**	
**Pill intake**	**Number of desired pills to take**	*“I: So how many pills a day do you think a person should take? R: One a day. I: Ok one right why do you think so? R: No people will get bored in taking two or three pills. (IDI, Female, 13–17 years, Cape Town)*	**One pill**	**13**	**NO**
		*“R: Maybe two. I: Two. R: Yes, in the morning and at night.(IDI, Male, 13–17 years, Soweto)*	**Two pills**	**8**	
**Alternative routes of administration**	**Method of PrEP administration**	*“P: Implant. I: Why? P: you do not have to go to the clinic if it is free. You don’t have to go to the clinic every time to get it” (IDI, Female, 18–24 years, Soweto)*	**Implant**	**6**	**NO**
		*“Since I am a forgetful person, I wish PrEP can be injectable just like contraception” (GD, Males and Females, 15–23 years, Cape Town)*	**Injection**	**20**	

#### Frequency of administration

The majority preferred a daily PrEP dosing regimen over an on-demand PrEP dosing regimen, citing unplanned sexual intercourse as one of the main reasons. Substance use was also identified as a factor that leads to unplanned sex.

*“The reason is because you don’t choose when you will have sex just like Esihle [pseudonym] mentioned that things such as Nandos, drugs and alcohol and the next thing you are kissing leading to sex.” (GD, Females, 18–24 years, Soweto)*

*“Because I don’t know when I’m going to have sex. I don’t plan to have sex it just happens.” (IDI, Female, 13–17 years, Cape Town)*

There were however some participants who preferred to take PrEP on demand stating that they might not remember to take a pill daily and and that it would be easier to take PrEP only when they know that they were going to engage in sexual intercourse.

*“I don’t see the reason why I should take it [PrEP] when I know I am not going to have sex, I am not going to be at risk.” (IDI, Male, 13–17 years, Cape Town)*

*“How am I going to remember to take the pill so I would rather take it [PrEP] as an on demand.” (GD, Females, 18–24 years, Soweto)*

#### Side effects

Some participants spoke of side effects from personal experience and some from information shared by people using PrEP. Participants stated multiple side effects, including, headaches, fatigue and “sleepiness”, that could be experienced when taking PrEP. They expressed that some side effects might only be experienced for a short period, such as nausea, vomiting, stomach aches and diarrhea, especially when the body is not used to the drug.

*“When you have just started maybe when you are not used to it [PrEP], maybe it can make you vomit but as you keep getting used to it, your body will get used to the idea that you are always taking tablets.” (GD, Females, 13–17 years, Soweto)*

*“For me, I heard that when you start something it has side effects, right? Other people said that this PrEP thing that you’re eating causes diarrhea, you see? Others become thin, others grow spots [a rash].” (GD, Males and Females, 13–20 years, Cape Town)*

Other potential side effects that participants felt could result from taking PrEP included changes of the skin, body weight, or developing a rash.

*“Side effects like if you take PrEP maybe you can gain weight. Or maybe you lose weight, do you see? Or maybe [change] complexion maybe it causes pimples.” (IDI, Male, 13–17 years, Cape Town)*

*“You might get a rash and you might think that you have an allergy.” (GD, Females, 18–24 years, Soweto)*

#### Cost of PrEP

The pricing of PrEP was based on GD and IDI participants’ suggestions. The actual cost of a months supply of daily generic Truvada (tenofovir-disoproxil and emtricitabine) as PrEP, as approximately R200 (~ 9GBP), was also taken into consideration when contextualizing the attribute-levels for the cost of PrEP. Some participants preferred PrEP to be free, while others expressed they would willing to pay. A few participants reported that they would not be able to access PrEP if there was a price attached to it due to a low socio-economic status. To buy PrEP would mean choosing between buying basic essential goods versus PrEP.

*“I agree with those who said we get it [PrEP] for free, some families are struggling financially and I live with my grandmother which means that from my granny’s money I have to get money for PrEP?” (GD, Females, 18–24 years, Soweto)*

*“I would prefer to get it [PrEP] for free.” (IDI, Male, 13–17 years, Cape Town)*

Participants suggested various prices of PrEP, ranging from R10 (50 cents) to a maximum of R300 (14GBP). The varying prices were also justified with the number of tablets that one person could afford and were also based on a monthly supply. Overall, participants stated it would be beneficial for them to be able to access PrEP to protect themselves against HIV, even when there is a cost attached to accessing PrEP.

*“P: I would be willing to even if okay for us especially in the hood [neighbourhood] there it could cost maybe R20 each I would meet means and hustle hard for them so that I know I am on a safe side. I: So R20 for a bottle? P: Each pill I: R20 for one pill.” (IDI, Male, 18–24 years, Soweto)*

*“R: R50. I: Okay you would buy it [PrEP] for R50 [confirms]. R: Uhm. I: And then how long would it [PrEP] last you? R: A month.” (IDI, Female, 18–24 years, Cape Town)*

#### Location of PrEP dissemination

It was important to get an understanding on where participants would like to access PrEP. Since services are free at the local clinics this is where some participants opted to access PrEP.

*“It will be easy for me to get it [PrEP] from the clinic because I don’t have money…, I don’t have money to buy it [PrEP], so that’s why I am saying the clinic. It will be easy for me to get it [PrEP] from the clinic.” (GD, Males, 13–17 years, Soweto)*

*“I would wish to get it [PrEP] from the clinic. Yes, a public clinic because at the clinic you are given pills, it is not known what these are for. They are handed out…when you go looking for a PrEP pill, you should receive it in a place for pills [pharmacy].” (IDI, Female, 18–24 years, Cape Town)*

Other participants opted for pharmacies, as they stated it was more private and confidential compared with a local clinic. Participants also stated that accessing PrEP through a pharmacy would be faster without long queues**.**

*“I would like to have it [PrEP] available at the pharmacy and be sold there. No one will see you when you go there {pharmacy] and no one knows you so you will just buy it [PrEP].” (GD, Males and Females, 15–23 years, Cape Town)*

#### Person responsible for dispensing PrEP

Participants stated that doctors, nurses and HIV counsellors would be best suited to dispense PrEP, because they were regarded as experienced and trained in testing and treating HIV, as well as being knowledgeable about prescribing and explaining PrEP to young people. However, some participants preferred a younger over an older health care provider to dispense PrEP.

*“I would prefer them [HIV counselors] because they are educated to counsel you first and make sure you are okay before you take something you don’t know. Nurses can explain but they don’t explain as well as a counsellor. A counsellor listens to you and you both listen to each other whereas a nurse just tells you what to do and they don’t even want to know about your concerns.” (GD, Females, 18–24 years, Soweto)*

*“A person for young people who is able to reach out to young people, someone friendly. So it may happen that she handles such department. She has to be young person so that we girls are able to talk to her and boys can have a male to talk to.” (GD, Males and Females, 15–23 years, Cape Town)*

#### PrEP dosing

Participants discussed different dosing periods for taking PrEP. These differed from the timeframe to take PrEP to be protected for the next sexual intercourse, to the time period PrEP should be taken once started on it.

*“I would prefer if they take it [PrEP] once a day but for a lifetime.” (IDI, Male, 18–24 years, Soweto)*

*“I may maybe say I would take it [PrEP] five days before [sex]. If I know that on that weekend I am going to be seen by my partner, something like that.”(IDI, Male, 13–17 years, Cape Town)*

***“****It [PrEP] should be ten years just like the loop so that we can see how well people take to it. Like this thing that they put in your arm.” (GD, Females, 18–24 years, Soweto)*

#### Pill intake

Overall, participants stated that they would be willing to take one pill or two pills per dose. However, the majority preferred to only take one pill, especially when taking it over a longer period of time. If taking two pills, participants would prefer to take one in the morning and the other in the evening.

*“One a day…people will get bored in taking two or three pills.” (IDI, Female, 13–17 years, Cape Town)*

*“It is easy to take one pill because if you take more than 1 pill the more you get discouraged to take it because you will be like so many pills why? Why do I have to take pills 3 times a day.” (IDI, Female, 18–24 years, Soweto)*

#### Alternative route of administration

During the GDs and IDIs, alternative routes of administration were discussed. Participants reported they would chose an implant or injectable as it would only need to be administered once and would not require returning to the clinic as frequently compared to using oral PrEP.

*“Since I am a forgetful person, I wish PrEP can be injectable just like contraception.” (GD, Males and Females, 15–23 years, Cape Town)*

*“Implant because injection people are scared of pain whenever we talking about injection comes pain in our heads but implant it is something which you do once off maybe then it last for a certain period which is intended for it to last.” (IDI, Male, 18–24 years, Soweto)*

### Expert opinion

**Fig. 1:**
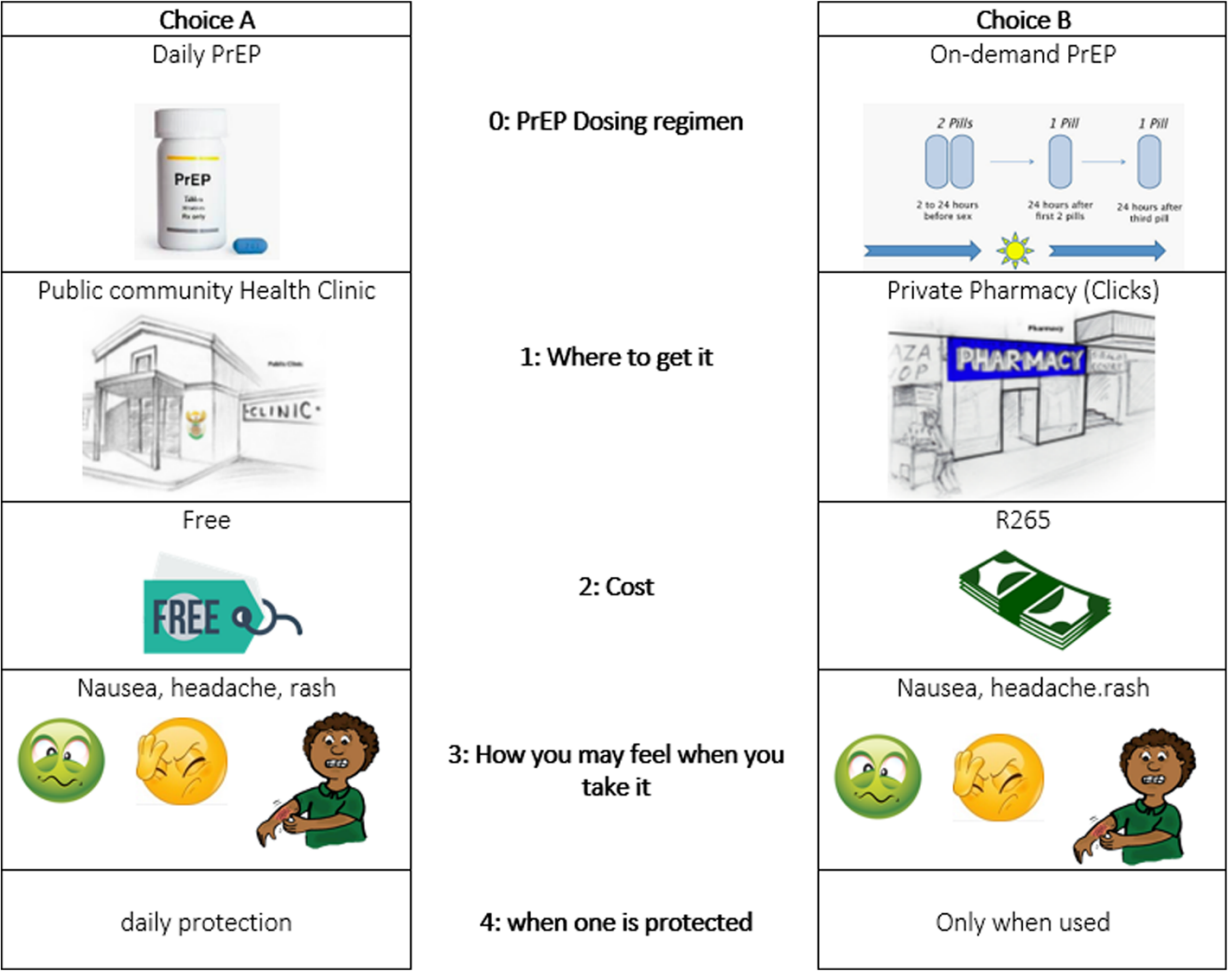
Example of a DCE choice set. Permission was granted from the iPrevent study team to utilize illustrations for public community health clinic and private pharmacy as part of our DCE choice sets [34]. For the other illustrations the following sources are acknowledged: Daily PreP [35]; on-demand PrEP [36]; cost illustrations [37, 38]; nausea [37]; headache [39]; rash [40]

The study team consulted two experts experienced in designing and implementing DCEs in South Africa. Through an iterative process of discussions with these experts four of the initial eight candidate attributes were retained with three attribute-levels, each, except for the dosing regimen attribute, which consisted of two attribute-levels, daily and on-demand PrEP. Table 1 indicates whether the attribute identified during the qualitative analysis process was retained after the discussion phase with the experts (last column). The discussion was aimed at selecting the most relevant attributes with a maximum of three attribute-levels each. This was also important to ensure the DCE incorporates a manageable number of attributes and level options to ensure that participants were not overwhelmed with the number of choice sets within the DCE. Consultation with other local team members ensured the language used for the attributes and attribute-levels was understandable and relevant within the South African context. For the location of PrEP dissemination it was deemed relevant to include a third attribute level, mobile clinic, as this has been a feasible approach to provide integrated health care, counselling services and dispensing of medication in underserved communities in Cape Town [34]. The final attributes and attribute-levels for the DCE included: dosing (daily and on-demand PrEP), location (private pharmacy, public clinic, mobile clinic), cost (free-of-charge, ZAR50 (~2GBP), ZAR200 (~ 9GBP)), and (4) side effects (nausea, headache, rash). After the iterative revision process, SAS was used to develop a statistically efficient and balances fractional factorial design of the DCE. Using SAS Enterprise Guide 7.15 (SAS Institute Inc., Cary, NC, USA) and the attributes identified in the qualitative study, the maximum possible number of attribute combinations was 2^3^ × 3^1^ [35]. A smaller factorial design was further developed using the D-Efficiency criteria reducing the choice sets to a feasible number of 6. The final DCE was constructed with a total of six binary choice sets with two scenario descriptions and graphics of the attribute-levels and participants can chose between Choice A and Choice B (Fig. 1).

Permission was granted from the iPrevent study team to utilize illustrations for public community health clinic and private pharmacy as part of our DCE choice sets [36]. For the other illustrations the following sources are acknowledged: Daily PreP [37]; on-demand PrEP [38]; cost illustrations [39, 40]; nausea [39]; headache [41]; rash [42].

### Pilot testing of the DCE with community advisory board members

To ensure a community engaged approach to the DCE development, the ACAB of the PHRU ratified the DCE choice sets as developmentally and contextually appropriate [20]. ACAB members regarded the number of choice sets as manageable, easy to understand, user-friendly and easy to follow. In addition, ACAB members agreed the illustrating graphics included in the DCE choice sets were complementary, relevant and context-specific for the attributes and related levels.

## Discussion

We explored PrEP delivery preferences among young people from a peri-urban setting in a multisite context in South Africa to develop a DCE. There are few methodological literature that describes the process to systematically infer attributes and its levels for a DCE, which is considered an important technique to better understand and provide guidance on user preferences for interventions and products [20, 22]. Previous research in South Africa and SSA that used a DCE focused on hypothetical HIV prevention methods [43], HIV testing, HIV and family planning services [21]. One multinational study conducted in seven countries among key user groups aged 16 - ≥41 years to elicited acceptability of PrEP and factors to determine uptake [44].

Even though DCEs are increasingly used in the field of sexual and reproductive health, there is limited research on young people’s PrEP preferences in developing countries. Young people are considered a high risk group of acquiring HIV infection and important beneficiaries in accessing PrEP, yet oral PrEP uptake is low amongst South African youth [17]. Amongst a sample of 772 young people aged 16–24 years in the Eastern Cape, South Africa, only 1.7% had used PrEP [17]. Once young people initiate on PrEP, a substantial proportion do not persist with it [45]. Our documentation of the DCE methodology provided a unique opportunity for targeted investigation on how to best implement PrEP amongst this population to support uptake in resource-limited settings.

During the qualitative interviews, young people expressed an initial preference for daily over on-demand PrEP. Participants stated that the use of daily PrEP would ensure protection for spontaneous or unanticipated sexual activities. Previous research shows that young people are often less likely to anticipate sex, which also includes communication and control within relationships [25]. This may be particularly true for young females, as they often have less decision making power and be less likely able to negotiate when sex occurs [46]. This might impact on chosing daily versus on-demand PrEP. In addition, young people are at high risk of non-adherence to medications, including contraception, ART or other chronic medications [47]. While it may be more difficult for young people adhering to a strict fixed pre-intercourse and/or post-intercourse PrEP dosing regimen, it may also impact on optimal adherence to a daily PrEP regimen.

Participants in our study discussed the tradeoffs between locations to access PrEP with costs and confidentiality. Participants opted for accessing PrEP at their local clinics, mainly because services would be free. Others preferred to access PrEP at a pharmacy, for ease of access, privacy and confidentiality. Stigma and discrimination are commonly known barriers to accessing public health care facilities and lead for young people to less likely engage in sexual and reproductive health care, including HIV services [48, 49]. Therefore, alternative locations for accessing PrEP might promote PrEP uptake among young people and support privacy and confidentiality, which was an important aspect for young people in our sample when getting PrEP. During a South African based demonstration project amongst females, PrEP initiation was high in youth-orientated, family planning and mobile outreach clinics [45]. Offering PrEP at different types of locations could be a suitable delivery model for young people. This may mitiage additional barriers, such as transportation, long distance to dispensing sites, and healthcare provider attitutes [50, 51]. In the South African setting, an integrated reproductive health care delivery model to access PrEP is feasible amongst females [45].

Participants in our study discussed their willingness to use alternative modalities for PrEP administration including injectables and implants. Current research is underway that tests the effectivenss of long-acting injectable formulations and intra-vaginal rings and potential alternatives to oral PrEP [52]. Further evaluations are required that focus on the willingness and perceptions of alternative application methods of PrEP amongst young people and not just for adults [53].

To better ensure that the final DCE was reflective of youth preferences we were able to incorporate a community engaged approach. The ACABs were actively involved in reviewing the choice sets for content, language, graphics and layout. This phase afforded the research team the opportunity to test the DCE choice sets supported response efficiency and content validity within the DCE [54, 55]. A youth engaged approach has been shown to work well in other settings, such as interventions for mental health, substance use and HIV prevention interventions [56, 57].

Next steps are to deliver the final DCE in a larger CHAPS survey and then to assess the preferences of young people for PrEP delivery in two South African cities, Soweto and Cape Town. It is anticipated that the results will provide important insights on how to align the implementation of PrEP to be most accessible and beneficial for young people, who present a key population in the HIV prevention response.

### Limitations

A few participants had prior PrEP experience, but the larger sample had limited to no prior exposure to PrEP. To avoid a potential bias based on those with PrEP experience, we included recruitment to locations outside of the clinic setting. Many participants that discussed topics related to PrEP access and use, drew from experience with other medication or from reports of others who took PrEP or where exposed to PrEP before. This was particularly true when they discussed the location to access PrEP and possible side effects. Limited prior knowledge on PrEP might have influenced participant’s responses. However, this was largely mitigated by the fact that discussions during the phase with experts confirmed the relevancy of the attributes and attribute-level identified during the qualitative interview phase.

At present, there is more biological evidence to support on-demand PrEP in males who have sex with males and less evidence to support such dosing options in females [58]. This qualitative research was conducted to explore participant preferences when provided with the choice for on-demand versus daily PrEP without explicitly stating that there was insufficient evidence for on-demand dosing in females. Previous research in adolescents showed a preference for using PrEP at periods of higher risk [59] – providing the impetus to better understand what it is that young people want. Therefore, our approach was to explore what ideal youth preferences were despite the biological evidence. Future research could explore how responses among young people would differ based on this biological limitation.

## Conclusions

There are few qualitative research methods studies describing the step-by-step process of developing a DCE for PrEP in adolescents, especially in resource-constrained countries. Using a systematic and robust approach, we use a developed and designed a DCE with attributes and levels for PrEP uptake that can be tested in young people in SA. We provide the process followed for the DCE technique to understand user preferences for daily and on-demand oral PrEP among young people in South Africa.

## Supplementary Information

**Additional file 1.** Demographic Questionnaire. The brief socio-demographic questionnaire included questions on sex, age, race, primary home language, sexual orientation, and education level.

**Additional file 2.** Qualitative Interview Guide for Group Discussions and In-Depth Interviews. A semi-structured guide including open ended questions and relevant probes to facilitate and guide the discussions during Group Discussions and In-Depth Interviews.

## Data Availability

The qualitative datasets generated and/or analysed during the current study are not publicly available to maintain participant privacy and confidentiality, but are available from the corresponding author on reasonable request.

## Abbreviations

ACAB: Adolescent Community Advisory Board
CHAPS: The Combined HIV Adolescent Prevention Study
DCE: Discrete Choice Experiment
DTHC: Desmond Tutu HIV Centre
FSW: Female sex worker
GDs: Group Discussions
HIV: Human Immunodeficiency Virus
IDIs: In-depth Interviews
IQR: Interquartile Range
MSM: Males who have sex with males
PHRU: Perinatal HIV Research Unit
PrEP: Pre-Exposure Prophylaxis
PWID: People who inject drugs
SA: South Africa
SPSS: Statistical Package for Social Sciences
SSA: sub-Saharan Africa
WHO: World Health Organization
