# Supplementary material for: A qualitative study to identify critical attributes and attribute-levels for a discrete choice experiment on oral pre-exposure prophylaxis (PrEP) delivery among young people in Cape Town and Johannesburg, South Africa
Source: BMC Health Serv Res. 2021 Jan 6;21:17. doi: 10.1186/s12913-020-05942-8 (PMC7788832; doi:10.1186/s12913-020-05942-8)
Supplement: Supplementary file 1 — Additional file 1. Demographic Questionnaire. The brief socio-demographic questionnaire included questions on sex, age, race, primary home language, sexual orientation, and education level. [file 12913_2020_5942_MOESM1_ESM.doc]

**Demographic Questionnaire**

**Study ID** ☐☐

**Please answer the following questions and remember that any information you provide will be kept confidential and no one will know it came from you. Please answer by marking (X) in the correct boxes or complete in text where necessary.**

1. **What is your sex?**

⬜ Male

⬜ Female

⬜ Transgender

⬜ Other (*please specify*): ________________

⬜ Prefer not to answer

1. **How old are you** (current age; please indicate age in years)? ___________

1. **How would you describe yourself (race)?**

⬜ Black African

⬜ Colored

⬜ Indian or Asian

⬜ White

⬜ Other (*please specify*): ___________________

⬜ Don’t know

⬜ Prefer not to answer

1. **What is your primary (main) home language:**

⬜ Afrikaans ⬜ IsiZulu ⬜ SiSwati

⬜ English ⬜ Northern Sotho ⬜ Tshivenda

⬜ IsiNdebele ⬜ Sesotho ⬜ Xitsonga

⬜ IsiXhosa ⬜ Setswana

⬜ Other (please specify): _____________

⬜ Prefer not to answer

1. **What is your sexual orientation**?
   ⬜ Heterosexual

⬜ Homosexual

⬜ Bisexual

⬜ Other (*please specify*): _____________

⬜ Don’t know

⬜ Prefer not to answer

1. **Which of the following best describes the housing that you currently live in? *Please select one.***

⬜ Brick house owned by family

⬜ Brick house that family is renting

⬜ Flat owned by family

⬜ Flat that family is renting

⬜ Reconstruction and Development Programme (RDP) house

⬜ Shack – Informal settlement

⬜ Shack – Backyard

⬜ Hostel

⬜ Outdoors, on the street, parks, or in a car

⬜ Other (*please specify*): ______________________

⬜ Don’t know

⬜ Prefer not to answer

1. **What is the highest level of education that you have completed?**

⬜ No schooling

⬜ Complete primary school (completed grade 7)

⬜ Incomplete primary school (up to grade 7)

⬜ Complete high school (completed grade 12)

⬜ Incomplete high school (up to grade 12)

⬜ Complete post-high school training (trade or technical training, college or university)

⬜ Incomplete post-high school training (trade or technical training, college or university)

⬜ Other *(please specify):* _________

⬜ Don’t know

⬜ Prefer not to answer
