# Supplementary material for: A qualitative study to identify critical attributes and attribute-levels for a discrete choice experiment on oral pre-exposure prophylaxis (PrEP) delivery among young people in Cape Town and Johannesburg, South Africa
Source: BMC Health Serv Res. 2021 Jan 6;21:17. doi: 10.1186/s12913-020-05942-8 (PMC7788832; doi:10.1186/s12913-020-05942-8)
Supplement: Supplementary file 2 — Additional file 2. Qualitative Interview Guide for Group Discussions and In-Depth Interviews. A semi-structured guide including open ended questions and relevant probes to facilitate and guide the discussions during Group Discussions and In-Depth Interviews. [file 12913_2020_5942_MOESM2_ESM.docx]

| **Qualitative Interview Guide for Group Discussion and In-Depth Interviews** |
| --- |
| **For Group Discussion:**  **Thank you for agreeing to be part of this group discussion. We will be talking about your perceptions on HIV risk and sexual risk behaviour among young people and the time we will spend together is approximately 1.5 hours. We will also discuss what you know about Pre-Exposure Prophylaxis (PrEP) and what you think will motivate or prevent young people to use it. Please feel free to ask me anything that is not clear to you regarding what we will be discussing and remember that there is no right or wrong answer.**  **For In-Depth Interviews:**  **Thank you for agreeing to be part of this interview. In the next hour we will be talking about your perceptions on HIV risk and sexual risk behaviour among young people. We will also discuss what you know about Pre-Exposure Prophylaxis (PrEP) and what you think will motivate or prevent young people to use it. Please feel free to ask me anything that is not clear to you regarding what we will be discussing and remember that there is no right or wrong answer.** |
| **PrEP in general**  What have you heard about Pre-exposure prophylaxis (PrEP) before today? Probe for who told them and what they heard about it.   - Please tell me about what you know about PrEP or about how it works. - How/where did you first learn about PrEP?     **If they have not heard about PrEP:**  Pre-Exposure Prophylaxis (PrEP) is a single pill that is a combination of two anti-HIV drugs, taken by HIV-negative people to reduce their risk of HIV infection. PrEP can either be taken orally daily for HIV prevention, or when needed (which is called on-demand PrEP), before and after sex. PrEP has been proven to be effective in reducing the risk of HIV infection. To be clear, PrEP protects you from HIV infection, not other STIs.     - Tell me what you think people in your community feel about taking medication every day to prevent HIV? - How would you feel about taking medication every day to prevent HIV infection? - How would you feel about taking medication only when you need it to prevent HIV infection? |
| **PrEP Characteristics**  ***Side effects***   - What do you think taking PrEP will do inside your body when you are HIV negative? - How do you think using PrEP may change anything in your life or in your daily life? - How do you think taking PrEP will affect your sexual experience?   ***Duration of taking PrEP (*days, weeks, years, lifetime)**   - What length of time do you think is reasonable for one to take PrEP?   ***Effectiveness***   - What percentage of effectiveness should PrEP have in order for people to use it? - How effective do you think PrEP is in preventing HIV infections?   (Interviewer to note to participant, if necessary, that PrEP is proven to be effective between 92-99%)    ***Frequency of administration (once a day vs. before sex acts)***   - Would you prefer taking PrEP daily? If so, why? - Would you prefer taking PrEP on-demand (before sex acts)? If so, why? - How many pills do you think are suitable to take daily? Why do you think so? - Would you prefer to get PrEP as an injectible/implant? If so, why?   ***Cost***   - Will you be willing to use PrEP if you were to pay for it? How much would you be willing to pay for PrEP? - Will you be willing to use PrEP if it was available for free?   ***Places of dissemination (pharmacy, clinic, adolescent/youth centre, doctor’s office)***   - Tell me about where you think you can get PrEP when you need to use it? - How easy do you think it will be for you to get PrEP? Tell me why you think   so.   - Where would you prefer to get PrEP?   ***Person who dispenses (doctors, nurses, peer counsellor, HIV counsellor)***   - Who would you prefer to give out PrEP for you and why? |
